# White matter hyperintensities and normal-appearing white matter integrity in the aging brain

**DOI:** 10.1016/j.neurobiolaging.2014.07.048

**Published:** 2015-02

**Authors:** Susana Muñoz Maniega, Maria C. Valdés Hernández, Jonathan D. Clayden, Natalie A. Royle, Catherine Murray, Zoe Morris, Benjamin S. Aribisala, Alan J. Gow, John M. Starr, Mark E. Bastin, Ian J. Deary, Joanna M. Wardlaw

**Affiliations:** a Brain Research Imaging Centre, Neuroimaging Sciences, University of Edinburgh, Edinburgh, UK; b Scottish Imaging Network: A Platform for Scientific Excellence (SINAPSE) Collaboration, Edinburgh, UK; c Centre for Cognitive Ageing and Cognitive Epidemiology (CCACE), University of Edinburgh, Edinburgh, UK; d Institute of Child Health, University College London, London, UK; e Department of Psychology, University of Edinburgh, Edinburgh, UK; f Department of Computer Sciences, Lagos State University, Lagos, Nigeria; g Department of Psychology, School of Life Sciences, Heriot-Watt University, Edinburgh, UK; h Alzheimer Scotland Dementia Research Centre, University of Edinburgh, Edinburgh, UK

**Keywords:** Aging, White matter hyperintensities, Normal-appearing white matter, Multimodal MRI

## Abstract

White matter hyperintensities (WMH) of presumed vascular origin are a common finding in brain magnetic resonance imaging of older individuals and contribute to cognitive and functional decline. It is unknown how WMH form, although white matter degeneration is characterized pathologically by demyelination, axonal loss, and rarefaction, often attributed to ischemia. Changes within normal-appearing white matter (NAWM) in subjects with WMH have also been reported but have not yet been fully characterized. Here, we describe the in vivo imaging signatures of both NAWM and WMH in a large group of community-dwelling older people of similar age using biomarkers derived from magnetic resonance imaging that collectively reflect white matter integrity, myelination, and brain water content. Fractional anisotropy (FA) and magnetization transfer ratio (MTR) were significantly lower, whereas mean diffusivity (MD) and longitudinal relaxation time (T1) were significantly higher, in WMH than NAWM (*p* < 0.0001), with MD providing the largest difference between NAWM and WMH. Receiver operating characteristic analysis on each biomarker showed that MD differentiated best between NAWM and WMH, identifying 94.6% of the lesions using a threshold of 0.747 × 10^−9^ m^2^s^−1^ (area under curve, 0.982; 95% CI, 0.975–0.989). Furthermore, the level of deterioration of NAWM was strongly associated with the severity of WMH, with MD and T1 increasing and FA and MTR decreasing in NAWM with increasing WMH score, a relationship that was sustained regardless of distance from the WMH. These multimodal imaging data indicate that WMH have reduced structural integrity compared with surrounding NAWM, and MD provides the best discriminator between the 2 tissue classes even within the mild range of WMH severity, whereas FA, MTR, and T1 only start reflecting significant changes in tissue microstructure as WMH become more severe.

## 1 Introduction

White matter hyperintensities (WMH) of presumed vascular origin are a common finding in brain magnetic resonance imaging (MRI) scans of healthy elderly individuals and are important features associated with impaired cognitive function in later life (Deary et al., 2003). These lesions appear as hyperintensities in the white matter on T2-weighted or fluid attenuated inversion recovery (FLAIR) MRI and have been described as white matter degeneration characterized by neuronal loss, demyelination, and gliosis on neuropathologic examination (Fazekas et al., 1993). Increasing age is one of the most important risk factors for WMH (Grueter and Schulz, 2012), although their underlying etiology is still unclear. They have been related to vascular risk factors (VRF; Grueter and Schulz, 2012) and linked to cerebral hypoperfusion or compromised blood-brain barrier integrity (Wardlaw et al., 2009) not only within visible lesions but also in the surrounding normal-appearing white matter (NAWM; O'Sullivan et al., 2002; Topakian et al., 2010; Uh et al., 2010). Additionally, it has been suggested that WMH severity, as seen on MRI, is an indirect marker of NAWM integrity (Maillard et al., 2011; Schmidt et al., 2010). However, it is unknown whether earlier compromised integrity of NAWM predates the appearance of WMH or the underlying pathology responsible for the lesions produces subtle changes in the surrounding NAWM which are invisible on conventional MRI. Structural changes in NAWM in people with WMH versus those without have been reported using a variety of imaging modalities, including structural, vascular, and metabolic MRI (Firbank et al., 2003; Maillard et al., 2011; Uh et al., 2010), although these changes are still to be fully characterized.

Structural changes in the integrity of the brain's white matter are commonly observed using diffusion tensor MRI (DT-MRI). Parameters obtained from the water diffusion tensor, such as fractional anisotropy (FA) and mean diffusivity (MD), can demonstrate alterations in axonal microstructure, with several studies showing that MD increases and FA decreases in areas of visible white matter degeneration such as that commonly observed in WMH (Bastin et al., 2009). Further potential MRI biomarkers of white matter damage are the magnetization transfer ratio (MTR) obtained from magnetization transfer MRI (MT-MRI) and the longitudinal relaxation time (T1). MTR can show pathologic alterations in white matter structure that involve macromolecules in the cell membrane, such as inflammation or demyelination, with decreased MTR being observed in WMH of presumed vascular origin (Fazekas et al., 2005). T1 provides quantitative information on brain water content and is therefore a potential marker for edematous brain tissue (Bastin et al., 2002). Differences in these imaging biomarkers can help identify the pathophysiological changes within NAWM and WMH in vivo; MTR and FA generally decline, and MD and T1 gradually increase during normal aging (Hsu et al., 2008, 2010; Silver et al., 1997). Because age is also strongly associated with the appearance of WMH, it could potentially confound the differences in WMH and NAWM integrity reported in previous studies, which typically include heterogeneous age groups. Changes observed in NAWM integrity relative to WMH load could also be a consequence of the older age of those subjects with more WMH, that is, a co-association between two features both associated with advancing age, rather than an effect of the lesions themselves or direct consequence of the same pathologic process.

In the present study, we used the previously mentioned imaging biomarkers to investigate microstructural differences between WMH and NAWM, as well as changes occurring in NAWM relative to WMH load, in a large cohort of community-dwelling older people, all born within the same year thereby minimizing the potential confounding effect of age. We hypothesized that the integrity of NAWM is influenced by the presence of WMH, independently of age or gender, because it is likely that white matter is diffusely affected by the processes that causes WMH even if the WMH only manifest themselves as visibly abnormal in discrete areas.

## 2 Methods

### 2.1 Participants

The Lothian Birth Cohort 1936 (LBC1936) comprises a group of subjects all born in 1936 and who are surviving participants of the Scottish Mental Survey of 1947. At recruitment in older age, the LBC1936 participants were predominantly community-dwelling individuals who agreed to follow-up cognitive and other medical and psycho-social assessments at approximately 70 years of age (Deary et al., 2007). During a second wave of this longitudinal study, at approximately 73 years of age, the LBC1936 participants also underwent comprehensive MRI to assess changes in brain structure (Wardlaw et al., 2011). Written informed consent was obtained from all participants under protocols approved by the National Health Service Ethics Committees.

### 2.2 Magnetic resonance imaging

All MRI data were acquired using a GE Signa Horizon HDxt 1.5 T clinical scanner (General Electric, Milwaukee, WI, USA) using a self-shielding gradient set with maximum gradient of 33 mT/m and an 8-channel phased-array head coil. The full details of the imaging protocol can be found in Wardlaw et al. (2011). Briefly, the MRI examination comprised whole-brain T1-weighted (T1W), T2-weighted (T2W), T2*-weighted (T2*W) and FLAIR-weighted structural scans, DT-MRI, MT-MRI, and T1 relaxation time mapping protocols. The DT-MRI protocol consisted of seven T2W (b = 0 s mm^−2^) and sets of diffusion-weighted (b = 1000 s mm^−2^) single-shot, spin-echo, echo-planar (EP) volumes acquired with diffusion gradients applied in 64 noncollinear directions (Jones et al., 2002). Two standard spin-echo sequences acquired with and without a magnetization transfer pulse applied 1 kHz from the water resonance frequency were collected for MT-MRI, whereas quantitative T1-mapping used two T1W fast-spoiled gradient echo sequences acquired with 2° and 12^o^ flip angles (Armitage et al., 2007). All sequences were acquired in the axial plane with a field-of-view of 256 × 256 mm, contiguous slice locations, and image matrices and slice thicknesses designed to give 2 mm isotropic voxels for DT-MRI and 1 × 1 × 2 mm (1 × 1 × 4 mm for FLAIR) voxel dimensions for the structural, MT-MRI, and T1-mapping protocols.

### 2.3 Visual scoring of white matter hyperintensities

A qualitative assessment of WMH load was performed by an expert neuroradiologist and cross-checked with a second consultant neuroradiologist, who scored hyperintensities in the FLAIR and T2W volumes using the Fazekas scale (Fazekas et al., 1987); a total score ranging from 0 to 6 was obtained by summing the periventricular and deep WMH Fazekas scores. To ensure observer reliability, one consultant neuroradiologist performed all the ratings after training on a standard data set. Another consultant neuroradiologist cross-checked a random sample of 20% of ratings, all scans with stroke lesions, and any scans where the first rater was uncertain. WMH were rated using the Fazekas scale as it is one of the most widely used visual rating scales and has been in use for over two decades. Fazekas scores are also closely correlated with quantitative measures of WMH volumes (Valdés Hernández et al., 2012).

### 2.4 Image analysis

All structural MRI volumes were registered to the corresponding T2W volume using linear registration (FMRIB's Linear Image Registration Tool; Jenkinson and Smith, 2001). Extracranial tissue was then excluded from each volume using brain binary masks obtained as described previously (Valdés Hernández et al., 2010).

**Fig. 1:**
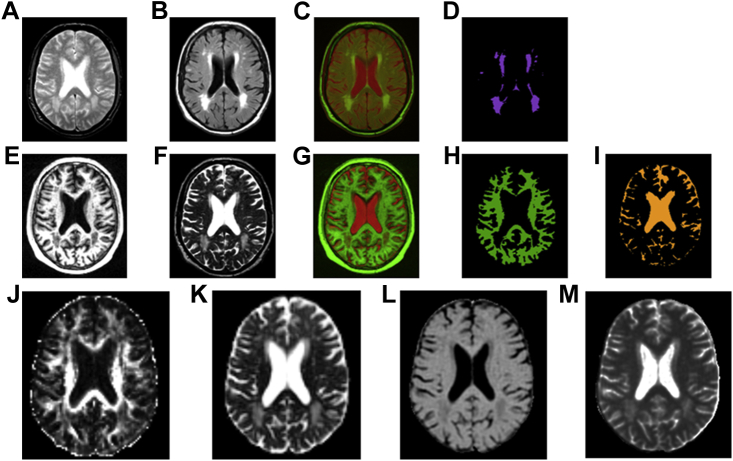
Multimodal MRI from a typical subject with WMH. T2*W (A) and FLAIR (B) structural scans are combined in red-green color space (C) to facilitate the extraction of WMH voxels (D). T1W (E) and T2W (F) structural scans are combined in red-green color space (G) to facilitate the extraction of NAWM (H) and CSF (I) voxels; the latter is subtracted from the WMH and NAWM masks to avoid CSF partial volume averaging within the measurement masks. The last row shows reconstructed parametric images of MRI biomarkers: FA (J), MD (K), MTR (L) and T1 relaxation time (M). Abbreviations: CSF, cerebrospinal fluid; FA, fractional anisotropy; FLAIR, fluid attenuated inversion recovery; MD, mean diffusivity; MRI, magnetic resonance imaging; MTR, magnetization transfer ratio; NAWM, normal-appearing white matter; WMH, white matter hyperintensity. (For interpretation of the references to color in this Figure, the reader is referred to the web version of this article.)

As shown in Fig. 1, NAWM and WMH tissue masks were obtained using the multispectral coloring modulation and variance identification (MCMxxxVI) method (Valdés Hernández et al., 2010). In brief, after registration of the T1W to the T2W volume from each data set, these volumes were mapped into red-green color space and fused; the minimum variance quantization clustering technique was then used in the resulting image to reduce the number of color levels, thereby allowing NAWM and cerebrospinal fluid (CSF) to be separated from other tissues in a reproducible and semi automatic manner. The same method was used to extract the WMH tissue mask from the T2*W and FLAIR volumes. Any stroke lesions (cortical, cerebellar, lacunes, and large subcortical) were identified by a neuroradiologist and excluded from the masks by hand by a trained image analyst.

DT-MRI volumes were preprocessed using FSL (http://www.fmrib.ox.ac.uk/fsl) to extract brain (Smith, 2002), remove bulk motion, and eddy current induced distortions by registering all subsequent volumes to the first T2W EP volume (Jenkinson and Smith, 2001) estimate the water diffusion tensor and calculate parametric maps of FA and MD from its eigenvalues using DTIFIT. Maps of MTR and T1 relaxation time were generated as described previously (Armitage et al., 2007; Silver et al., 1997).

For each data set, linear registration (Jenkinson and Smith, 2001) was used to ensure accurate correspondence between the parametric maps and the space of the tissue masks (T2W). FMRIB's Linear Image Registration Tool was applied with 6 degrees of freedom to the MTR and T1 maps to correct for bulk motion and 12 degrees of freedom (affine) between the structural and diffusion T2W volumes to obtain the transformation of the tissue masks into diffusion space. To avoid small partial volume averaging with CSF because of registration inaccuracies, the CSF mask was dilated by 1 voxel in each direction and then subtracted from the NAWM and WMH masks. After registration, the binary WMH and NAWM masks were used to obtain averaged FA, MD, MTR, and T1 values for these tissues in each participant.

### 2.5 Spatial relationship between WMH and NAWM

We assessed how the proximity of the WMH affected the NAWM integrity using a region-of-interest (ROI) analysis with regions drawn at a range of distances from the WMH. To create these ROI for each participant we dilated the WMH masks by increments of 2 mm up to 10 mm, then subtracted from each dilated ROI the previous one, that is, the WMH mask was subtracted from the 2 mm ROI, the 2 mm ROI subtracted from the 4 mm ROI, and so on, so only the surrounding contours remained (the distances quoted are approximate as they are limited by finite voxel size). These ROIs were also created in diffusion space. To avoid running into tissues other than NAWM, we kept only those voxels in each ROI which intersected with the NAWM mask. An example is shown in Supplementary Fig. 2. For each subject we measured the averaged parameters within each of the new ROI, as well as within the remaining NAWM and plotted these data (corrected by age in days and gender) for each distance to assess the spatial relationship with WMH. To demonstrate that the anatomic location of the WMH does not affect the biomarkers measured in NAWM, we also performed an analysis using small ROI placed in exactly the same locations in all participants (Supplementary Material).

### 2.6 Statistical analysis

Significant differences between averaged FA, MD, MTR, and T1 values in WMH and NAWM were tested using paired *t* tests, with effect sizes assessed using Cohen d. Logistic regression and receiver operating characteristic (ROC) curve analysis was performed to assess which parameter independently discriminated best between WMH and surrounding NAWM and its prediction value, using the masks obtained with the semiautomatic method as ground truth. Averaged FA, MD, MTR, and T1 values in NAWM were compared across the seven categories of total Fazekas score (0–6) using analysis of covariance (ANCOVA) with gender and age in days at the time of scanning as covariates. Images from outliers in the data were inspected visually and discarded from the analysis if the outlying value was caused by the image acquisition or processing problems, such as motion or failed registration.

All analyses were performed using the R software environment for statistical computing (R Development Core Team, 2012; Tabelow et al., 2011), along with the “pROC,” “car,” “effects,” and “ggplot2” packages (Fox, 2003; Fox and Weisberg, 2011; Robin et al., 2011; Wickham, 2009).

### 2.7 Vascular risk factors

The analysis for NAWM was repeated using self-reported history of smoking (current, ex-, and non-smoker), hypertension, hypercholesterolemia, diabetes, cardiovascular disease, and stroke (either self-reported or evident on MRI) as covariates in the ANCOVA to adjust for potential effects of VRF in the measured imaging parameters or their association with Fazekas score. Attenuation of any statistical difference in the measured imaging parameters between the Fazekas score groups could suggest confounding by these factors or mediation. To test the relevance of VRF in the extended model, the nested models were compared with and without including the VRF using the F-test.

## 3 Results

Among the 700 participants who underwent MRI, 24 were excluded because of incomplete imaging data leaving a total of 676 subjects (358 men and 318 women); the mean age at time of scanning was 72.7 ± 0.7 years (range, 71.0–74.2 years). Gender and age details for each total Fazekas score group are reported in Table 1.

### 3.1 White matter hyperintensities versus normal-appearing white matter

**Fig. 2:**
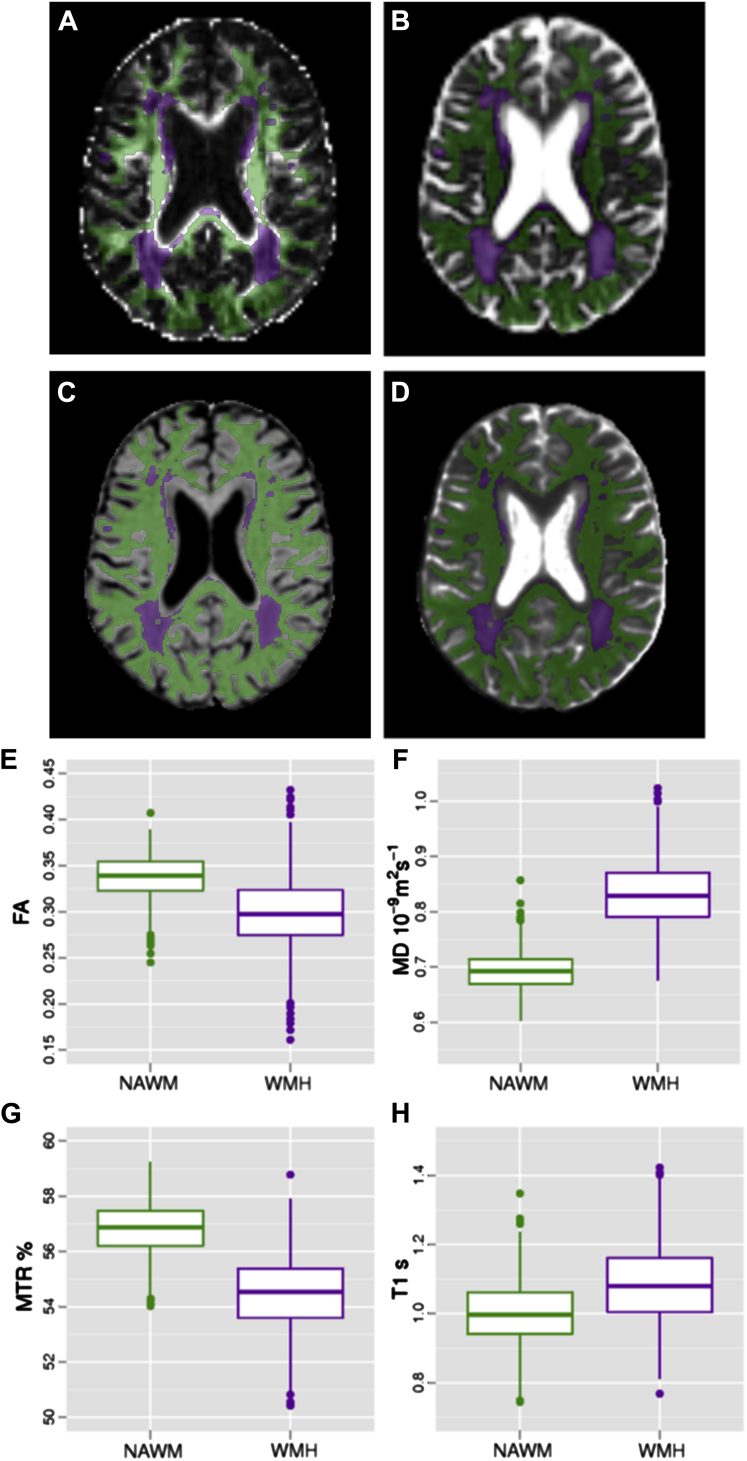
Example of NAWM (green) and WMH (magenta) masks overlaid onto the MRI parametric maps for a typical subject, and the corresponding box plots for the average values of each parameter measured in both tissue types across the cohort: (A) and (E) for FA, (B) and (F) for MD, (C) and (G) for MTR and (D) and (H) for T1 relaxation time. Abbreviations: FA, fractional anisotropy; MD, mean diffusivity; MRI, magnetic resonance imaging; MTR, magnetization transfer ratio; NAWM, normal-appearing white matter; WMH, white matter hyperintensity. (For interpretation of the references to color in this Figure, the reader is referred to the web version of this article.)

Fig. 1 shows multimodal MRI from a typical participant presenting with WMH. Values of FA and MTR were significantly lower whereas MD and T1 were significantly higher in WMH than NAWM (*p* < 0.0001), with MD providing the largest difference between the two tissue classes (Fig. 2 and Table 2); all effect sizes were large. In all box plots, the boxes represent the lower and upper quartiles and the median measurement (thick line) for each group. Whiskers indicate the sample minimum and maximum, whereas the represented outliers (dots) differ from the lower and upper quartiles by more than 1.5 times the interquartile range.

**Fig. 3:**
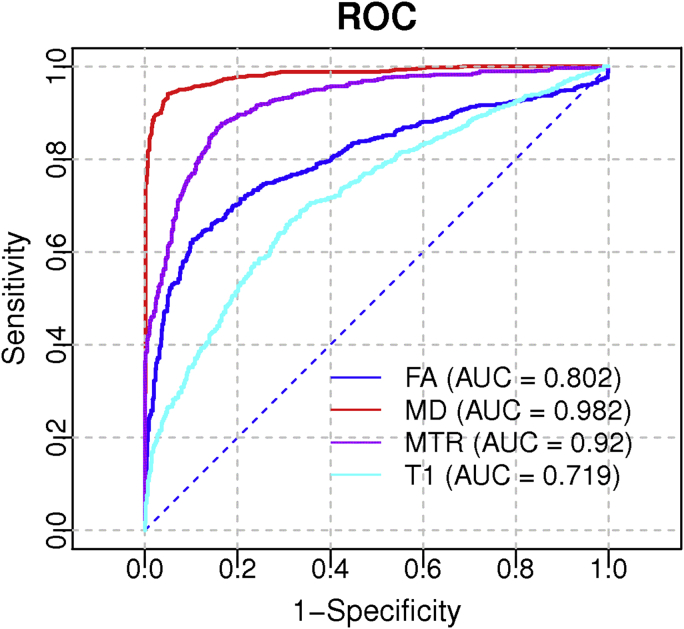
ROC curves showing the ability to discriminate between NAWM and WMH for the four imaging biomarkers. AUC values are shown on the legend. Abbreviations: AUC, area under the curve; NAWM, normal-appearing white matter; ROC, receiver operating characteristic; WMH, white matter hyperintensity. (For interpretation of the references to color in this Figure, the reader is referred to the web version of this article.)

Logistic regression on each individual parameter confirmed that MD differentiated best between NAWM and WMH. ROC analysis produced an optimal threshold of 0.747 × 10^−9^ m^2^s^−1^ for MD, with 0.95 specificity and 0.94 sensitivity, to discriminate 94.6% of the lesions. The effect of varying this threshold is shown in the ROC curve (Fig. 3), with an area under the curve of 0.982 for MD (95% CI: 0.975–0.989), which is significantly higher than the area under the curve obtained with FA, MTR, or T1.

### 3.2 Normal-appearing white matter integrity changes with Fazekas score

**Fig. 4:**
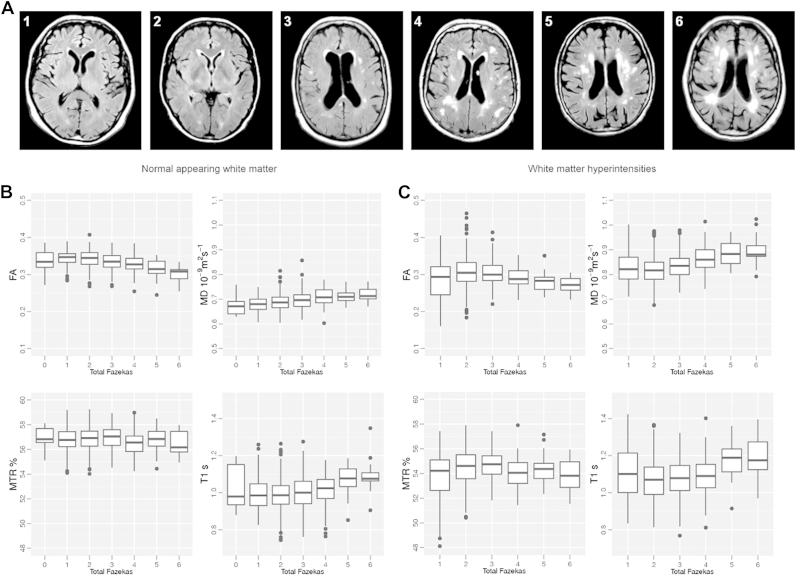
Brains were classified according to their WMH load; periventricular (0–3) and deep (0–3) WMH scores were summed, with total Fazekas scores ranging from 0 (no visible WMH) to 6 (widespread WMH). (A) Examples of brains within the range of total Fazekas scores (score shown in left top corner of each axial image). Box plots of the averaged FA, MD, MTR, and T1 relaxation time measured for each total WMH load score in NAWM (B) and WMH (C). Abbreviations: FA, fractional anisotropy; MD, mean diffusivity; MRI, magnetic resonance imaging; MTR, magnetization transfer ratio; NAWM, normal-appearing white matter; WMH, white matter hyperintensity.

There were significant differences in NAWM imaging biomarkers across the total Fazekas score groups as indicated by ANCOVA (Fig. 4 and Table 3). There were significant decreases in FA and MTR and significant increases in MD and T1 with increasing total Fazekas score (Table 3). The total Fazekas score, together with age and gender as covariates, explained 16% and 13% of the variance in FA and MD, respectively, and 9% of the variance in MTR and T1 in NAWM.

As shown in Table 1, the group of subjects with a Fazekas of zero was small (N = 9), and this can potentially affect the accuracy of average biomarkers in this group (as it is reflected by the larger SD shown in Table 3).

### 3.3 Vascular risk factors

The ANCOVA was repeated with self-reported VRF as covariates. The reported incidence of each of the factors, as well as the incidences in male and female, is reported in Table 4. The incidence of ex-smoking, diabetes, and cardiovascular disease was significantly different between genders (*p* < 0.01). Including VRF increased the variance explained by the model in all four biomarkers as expected (see Table 3, last row). The significant associations between imaging biomarkers and the total Fazekas score were not attenuated by the addition of these potential confounding variables in the model. However, to test the relevance of VRF, the nested models were compared with and without including them. There were significant differences in the models with and without VRF for FA and MD (F = 1.5; *p* = 0.02 in both) and a trend for T1 (F = 1.3; *p* = 0.09) but no significant difference for MTR (F = 1.2; *p* = 0.22).

### 3.4 Spatial relationship between WMH and NAWM

**Fig. 5:**
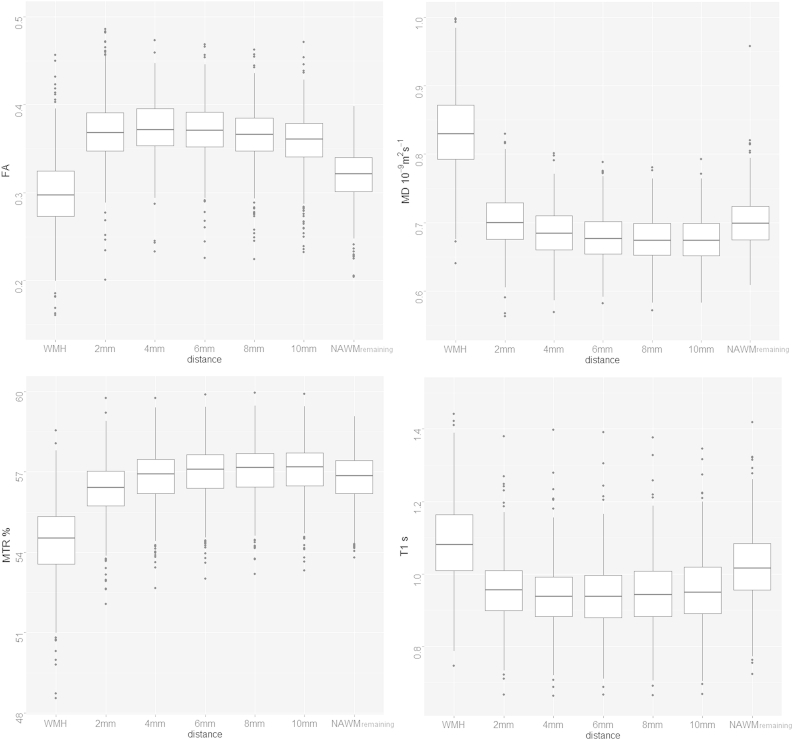
Box plots of NAWM measured in ROI contouring the WMH for all subjects at different (approximate) distances between 2 mm and 10 mm, as well and the remaining NAWM. The data for WMH are included for reference. Abbreviations: NAWM, normal-appearing white matter; ROI, region of interest; WMH, white matter hyperintensity.

**Fig. 6:**
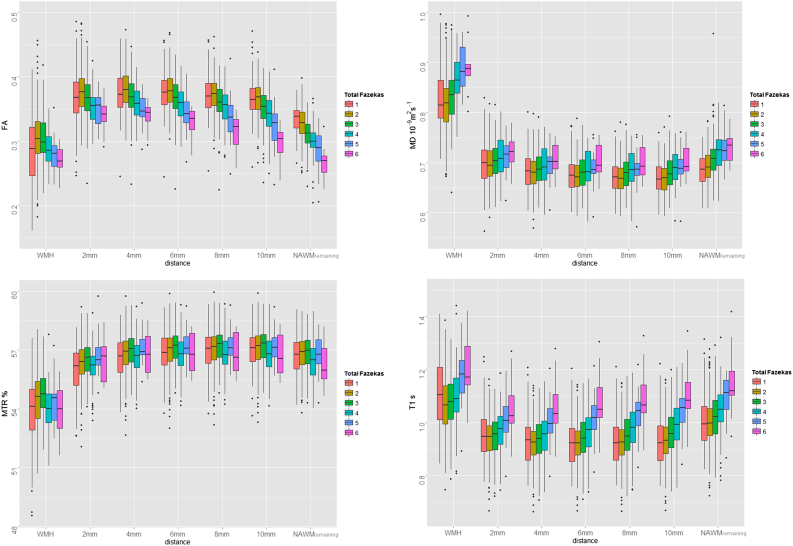
Box plots of NAWM measured in ROI contouring WMH at (approximate) distances between 2 mm and 10 mm, as well and the remaining NAWM, with data divided by Fazekas score as indicated by different color boxes. The data for WMH are included for reference. Abbreviations: NAWM, normal-appearing white matter; ROI, region of interest; WMH, white matter hyperintensity. (For interpretation of the references to color in this Figure, the reader is referred to the web version of this article.)

Fig. 5 shows the box plots of the imaging biomarkers measured in NAWM at a range of distances from the WMH for all participants. Data for the WMH are included for reference. Both MD and T1 decrease with distance from the WMH, whereas MTR increases. FA shows a slight increase when moving from 2 mm to 4 mm; however, it decreases thereafter. Fig. 6 shows the same data divided by total Fazekas scores; the changes with distance are the same as in Fig. 5 for all Fazekas scores. The pattern of changes of NAWM biomarkers with lesion load also does not vary with distance, as the pattern shown in Fig. 4B remains consistent for the ROI surrounding the WMH, as shown in Fig. 6. The analysis of the small ROI that sampled the same small points in NAWM of each participant confirmed that change in NAWM with Fazekas score was not simply because of variation in underlying FA by location of remaining NAWM (Supplementary Material).

## 4 Discussion

Results from the four imaging biomarkers used in the present study indicate that WMH have reduced white matter integrity compared with NAWM, with the integrity of NAWM, in turn, affected by the severity of these lesions even after accounting for age, gender, and self-reported VRF. The effect of lesion severity in NAWM was also independent of proximity to WMH. The difference in microstructure is reflected, in particular, by MD which provides the best discriminator between WMH and NAWM, correctly identifying 94.6% of the lesions. The significant increase in MD observed within WMH is also accompanied by an increase in T1, although the narrow range of values that this latter parameter takes limits its value in discriminating between NAWM and WMH. FA and MTR also provide less discrimination between the two tissue classes than MD, although they are still significantly reduced in WMH compared with NAWM. These results agree with previous reports of compromised NAWM in the presence of WMH using different quantitative imaging techniques (Firbank et al., 2003; Taylor et al., 2007; Topakian et al., 2010; Uh et al., 2010; Vernooij et al., 2008), although most studies used small cohorts, included subjects with a wide age range, and did not measure the variety of imaging biomarkers acquired here. The present study's observations indicate that brain tissue pathology spreads beyond the area of visible WMH. Furthermore, the spatial analysis shows that changes to NAWM are locally dependent on distance from the WMH in agreement with previous reports (Maillard et al., 2011). In our analysis however, FA decreases slightly with distance rather than increasing as it would be expected. A likely explanation for this is the location of the WMH; they generally appear in, or close to, areas of the brain with long association and commissural white matter tracts (see Supplementary Fig. 1), and hence, the ROI surrounding them will have FA higher than that averaged over the whole NAWM. This location “effect” also explains why the remaining NAWM tissue (Figs. 5 and 6) shows slightly decreased FA and MTR, and increased MD and T1, when compared with ROI immediately surrounding the lesions.

To test further the observation that changes in NAWM with Fazekas score were not simply the effect of WMH location, we further corroborated these results using small ROI measured in exactly the same locations for all participants. This analysis demonstrated that these findings were not simply a function of differential white matter integrity between regions affected and not affected by WMH (see Supplementary Material), something not considered in previous studies. We repeated the ANCOVA after adjustment for common VRF, nested models with and without risk factors, which did not alter the overall results and demonstrated the robustness of our approach measuring biomarkers averaged over the whole NAWM.

The precise sequence of pathologic processes underpinning the microstructural changes in white matter integrity within and around WMH are yet to be fully described. Pathology reports have emphasized the rarefaction, demyelination, and axonal loss in tissue corresponding to WMH, but of necessity, this is often in late-stage disease. These changes have been attributed to ischemia, which can lead to the rarefied appearance of white matter (Wardlaw et al., 2013b) and could cause the observed increases in MD and T1, and the decreases in FA and MTR seen in established WMH. However, the increase in MD in NAWM even with the mildest Fazekas score suggests that altered water mobility in the interstitial space may be an early feature of white matter pathology in the aging brain. A chronically although subtly compromised blood-brain barrier, as occurs with advancing age (Farrall and Wardlaw, 2009), leading to increased fluid in the extracellular space and perivascular tissue damage (Lammie et al., 1998) could also produce the observed changes in these imaging biomarkers (Wardlaw et al., 2013b). WMH load was also an independent predictor of increased blood-brain barrier permeability in NAWM in previous studies, consistent with leakage preceding the development of WMH and thus playing a causal role (Farrall and Wardlaw, 2009; Topakian et al., 2010; Wardlaw et al., 2009). Further work relating these imaging biomarkers to histopathologic findings especially at early stages in disease is required to understand fully the pathologic processes, which are responsible for white matter damage within and around WMH.

In addition to the narrow age range of our subjects, which minimizes the confounding effects of age-related change in MD, FA, MTR, and T_1_, further strengths of the study include the large sample size, the use of imaging, and analysis methods which conform to STRIVE standards (Wardlaw et al., 2013a) and recruitment from a single centre which removes multicentre effects. We also measured the integrity of whole-brain NAWM, removing bias which could result from the use of predefined ROI and has proven to be a robust method regarding potential effects of WMH location, something not considered in previous reports. A weakness of this study is potential partial averaging of CSF within the measurement masks, particularly as linear registration was used to align the T2W and the DT-MRI volumes. However, this problem was mitigated by dilating the CSF masks by 1 voxel in all directions at the processing stage and by rejecting any outlying data caused by registration before statistical analysis. Excluding 1 voxel around the CSF mask should be sufficient to reduce significantly the CSF contamination from the measurement masks as this would only affect those voxels that fall right at the ventricle and sulcus boundary. Finally, the threshold values for discriminating WMH from NAWM derived from the ROC analysis in this cohort need to be confirmed in further studies of normal aging, ideally in populations with narrow age range.

In summary, the present study indicates that WMH have reduced structural integrity compared with surrounding NAWM, with the integrity of NAWM, in turn, reflecting the severity of WMH. These multiparameter in vivo observations suggest that changes in white matter microstructure are reflected most strongly by MD which provides the best discriminator; in NAWM, this biomarker shows changes even within the mild range of WMH severity, whereas the other 3 biomarkers only start reflecting changes as WMH become more severe. The exceptional discriminant value of 94.6% of the lesions that was obtained with MD has not been reported previously and could reflect the near-homogeneous ages of the participants of this study, or the fact that MD is more sensitivity to age-related changes in tissue microstructure than the other three biomarkers (Maclullich et al., 2009). The cohort used in the study are currently undergoing a second wave of MRI thus providing an opportunity to investigate the roles that current and prior levels of MRI-derived NAWM integrity and risk factors play in the progression of white matter damage and its consequences for healthy aging.

## Disclosure statement

The authors declare that they have no actual or potential conflicts of interest with this work.

## Figures and Tables

**Table 1:** Description of the study population by total Fazekas score for WMH

Total Fazekas score	N	N male (%)	Mean age (SD)
0	9	6 (67)	72.7 (0.4)
1	100	57 (57)	72.5 (0.7)
2	311	161 (52)	72.7 (0.8)
3	143	86 (60)	72.6 (0.7)
4	72	32 (44)	72.9 (0.6)
5	28	13 (46)	72.7 (0.6)
6	13	3 (23)	72.7 (0.6)

Periventricular (0–3) and deep (0–3) WMH scores were summed. Key: SD, standard deviation; WMH, white matter hyperintensity.

**Table 2:** Results of *t* tests comparing the averaged imaging parameters measured within areas of NAWM and WMH

Mean values	NAWM	WMH	*p*	Cohen d
FA	0.338 ± 0.024	0.299 ± 0.042	<0.0001	1.13
MD (10^−9^m^2^s^−1^)	0.692 ± 0.034	0.833 ± 0.061	<0.0001	−2.85
MTR (%)	56.80 ± 0.99	54.37 ± 1.51	<0.0001	1.91
T1 (s)	1.002 ± 0.092	1.086 ± 0.117	<0.0001	−0.80

Effect sizes are shown as Cohen d. Key: FA, fractional anisotropy; MD, mean diffusivity; MTR, magnetization transfer ratio; NAWM, normal-appearing white matter; WMH, white matter hyperintensity.

**Table 3:** Mean (SD) values of FA, MD, MTR, and T1 relaxation time measured in NAWM for each total Fazekas score WMH group

Total Fazekas score	FA	MD 10^−9^m^2^s^−1^	MTR %	T1 s
0	0.336 (0.037)	0.675 (0.042)	56.9 (1.0)	1.02 (0.12)
1	0.343 (0.020)	0.681 (0.031)	56.7 (1.1)	1.00 (0.10)
2	0.342 (0.023)	0.688 (0.033)	56.8 (0.9)	0.99 (0.09)
3	0.335 (0.022)	0.698 (0.035)	57.0 (0.9)	1.00 (0.09)
4	0.328 (0.023)	0.709 (0.034)	56.5 (1.0)	1.01 (0.09)
5	0.314 (0.026)	0.712 (0.028)	56.8 (1.0)	1.07 (0.07)
6	0.299 (0.025)	0.720 (0.029)	56.5 (1.1)	1.09 (0.11)
Gender + age				
F	16.2	8.9	2.3	6.0
*p*	<0.0001	<0.0001	0.03	<0.0001
R^2^	0.16	0.13	0.09	0.09
Gender + age + VRF^a^				
F	14.5	8.6	2.4	5.7
*p*	<0.0001	<0.0001	0.02	<0.0001
R^2^	0.25	0.22	0.17	0.17

Last rows present the F, *p*, and R^2^ values from ANCOVA, using only gender and age as covariates and including also VRF. Key: ANCOVA, analysis of covariance; FA, fractional anisotropy; MD, mean diffusivity; MTR, magnetization transfer ratio; NAWM, normal-appearing white matter; WMH, white matter hyperintensities; VRF, vascular risk factors.

a Self-reported smoking, hypertension, diabetes, hypercholesterolemia, cardiovascular disease, and stroke.

**Table 4:** Incidence of self-reported VRF

Vascular risk factor	N	Total (%)	Male (%)	Female (%)	*p*
Current smoker	56	8.1	7.8	8.5	0.75
Ex-smoker	314	45.4	50.8	38.7	*<0.01*
Hypertension	339	49.1	50.8	46.9	0.30
Diabetes	75	10.9	13.1	6.6	*<0.01*
Hypercholesterolemia	287	41.5	42.5	40.9	0.68
Cardiovascular disease	188	27.2	33.2	19.8	*<0.01*
Stroke	123	17.8	18.2	15.6	0.85

Significant differences between male and female incidence of VRF are determined by Pearson χ^2^ test. Italic indicates statistical significance *p* < 0.05.
