# Supplementary material for: White matter hyperintensities and normal-appearing white matter integrity in the aging brain
Source: Neurobiol Aging. 2015 Feb;36(2):909–18. doi: 10.1016/j.neurobiolaging.2014.07.048 (PMC4321830; doi:10.1016/j.neurobiolaging.2014.07.048)

White matter hyperintensities and normal-appearing white matter integrity in the ageing brain

Susana Muñoz Maniega^a,b,c^; Maria C. Valdés Hernández^a,b,c^; Jonathan D. Clayden^d^; Natalie A Royle^a,b,c^; Catherine Murray^c,e^; Zoe Morris^a,b^; Benjamin S. Aribisala^f^; Alan J. Gow^c,g^; John M. Starr^c,h^; Mark E. Bastin^a,b,c^; Ian J. Deary^c,e^; Joanna M. Wardlaw^a,b,c^

^a^Brain Research Imaging Centre, Neuroimaging Sciences, University of Edinburgh, Edinburgh, UK.

^b^Scottish Imaging Network, A Platform for Scientific Excellence (SINAPSE) Collaboration, Edinburgh, UK.

^c^Centre for Cognitive Ageing and Cognitive Epidemiology (CCACE), University of Edinburgh, Edinburgh, UK.

^d^Institute of Child Health, University College London, London, UK.

^e^Department of Psychology, University of Edinburgh, Edinburgh, UK.

^f^Department of Computer Sciences, Lagos State University, Lagos, Nigeria

^g^Department of Psychology, School of Life Sciences, Heriot-Watt University, Edinburgh, UK

^h^Alzheimer Scotland Dementia Research Centre, University of Edinburgh, Edinburgh, UK

8^th^ July 2014

**Correspondence:**

Dr ME Bastin ([Mark.Bastin@ed.ac.uk](mailto:Mark.Bastin@ed.ac.uk)) or Prof JM Wardlaw ([jwardlaw@staffmail.ed.ac.uk](mailto:jwardlaw@staffmail.ed.ac.uk))

Brain Research Imaging Centre, Neuroimaging Sciences, University of Edinburgh, Western General Hospital, Crewe Road, Edinburgh, EH4 2XU, UK.

**Supplementary Material**

# Region-of-interest analysis of the effects of Fazekas score on normal-appearing white matter integrity

Since areas of visible WMH were discarded from the NAWM measurements, averaged values of the imaging biomarkers obtained from this tissue could be affected by the typical location of WMH. For example, since WMH commonly develop in areas of white matter with high FA (such as periventricular white matter), excluding these areas from the measurement mask could produce a lower FA on average in the remaining tissue, an effect that would increase the larger the area of WMH that is excluded. This could produce an artefactual reduction in NAWM integrity with increasing WMH load. An ROI analysis was therefore performed to test the reliability of changes detected in NAWM associated with WMH load.

The ROI analysis was performed by first identifying a representative brain from the sample in terms of volume, atrophy and WMH load. Averaged maps of NAWM and WMH were created by non-linear registration of every subject to the representative brain using Niftyreg ([http://sourceforge.net/projects/niftyreg](http://sourceforge.net/projects/niftyreg/)). Eight small ROI (4 × 4 mm) were placed in areas of the averaged NAWM all of which fell outside the averaged WMH map, i.e. no subject provided lesions in those areas which corresponded to genu and splenium of corpus callosum, corticospinal tracts and prefrontal and occipital white matter external to any periventricular WMH (see Supplementary Figure 1). These ROI were then transferred to each subject’s native space using non-linear registration where mean FA, MD, MTR and T1 values were obtained. ROI were discarded if, due to small registration errors, they overlapped with the WMH mask of the individual in native space.

Averaged FA, MD, MTR and T1 values in NAWM were compared across the seven categories of total Fazekas score (0 to 6) using analysis of covariance (ANCOVA) with gender and age in days at the time of scanning as covariates using data measured in the ROI.

In 13 cases, ROI were discarded due to overlap with the subject’s WMH mask. The ROI analysis confirmed the results obtained from the biomarkers measured in the whole NAWM masks (Table 3). For the whole group, there was a significant decrease in FA (F_6,627_ = 3.6; *p* = 0.002), a trend for decrease in MTR (F_6,607_ = 2.0; *p* = 0.06) and significant increases in both MD and T1 (F_6,627_ = 3.7; *p* = 0.001 and F_6,648_ = 6.2; *p*< 0.0001) with increasing total Fazekas score. As these parameters were measured in exactly the same areas of NAWM, this demonstrates that the changes observed for whole NAWM are not just a consequence of the location of the WMH.

**Figure 1:** Averaged maps of NAWM (green) and WMH (magenta) over a study-representative brain. The slices show the square ROI (yellow) used for verification of the results obtained with the NAWM masks.

*
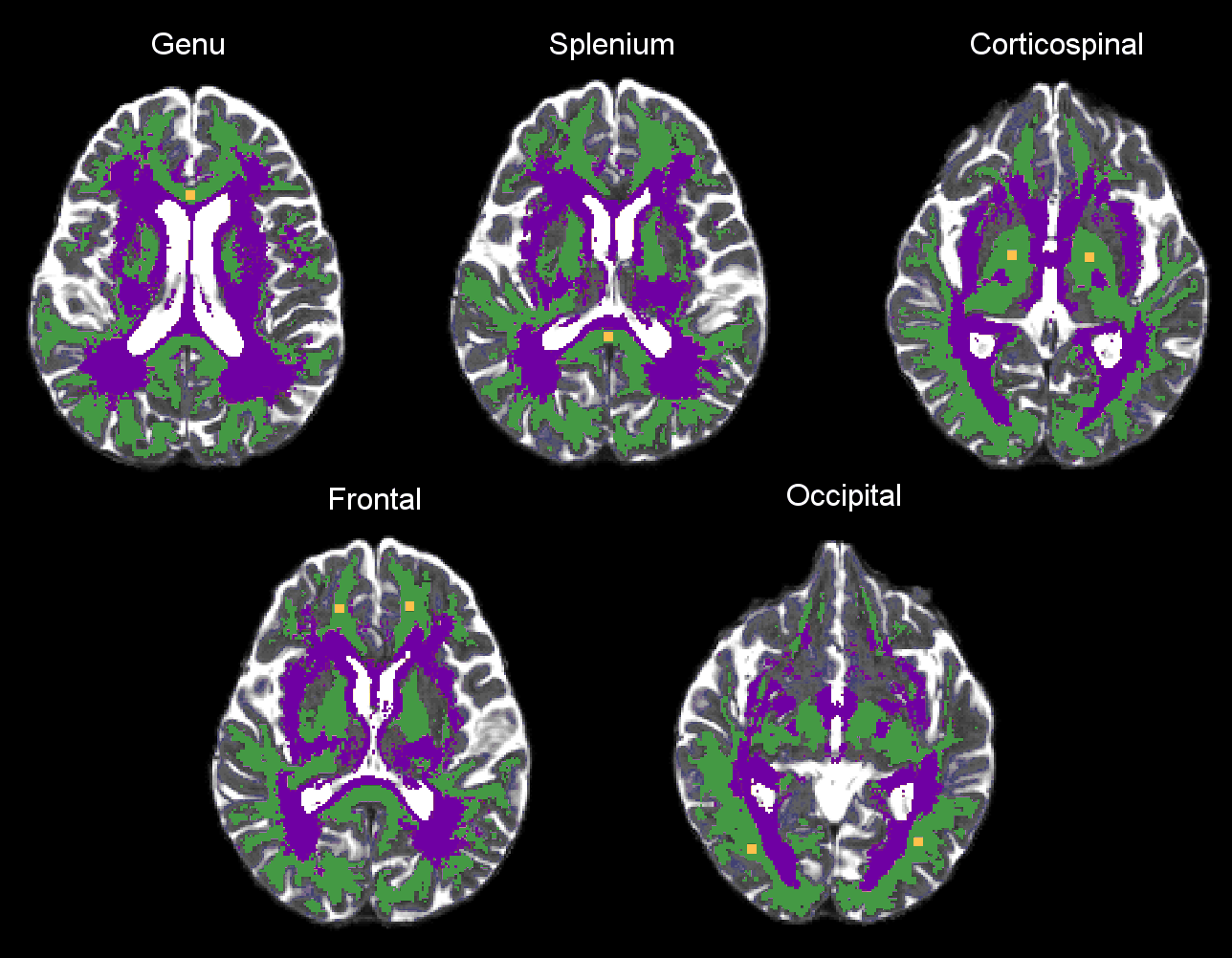
*

**Figure 2:** Example of ROI used for the analysis of spatial relationships between WMH (bright red) and NAWM (dark green) in a T2W volume. Contours are 2 voxels thick forming ROI at approximately 2, 4, 6, 8 and 10 mm from the WMH edge.


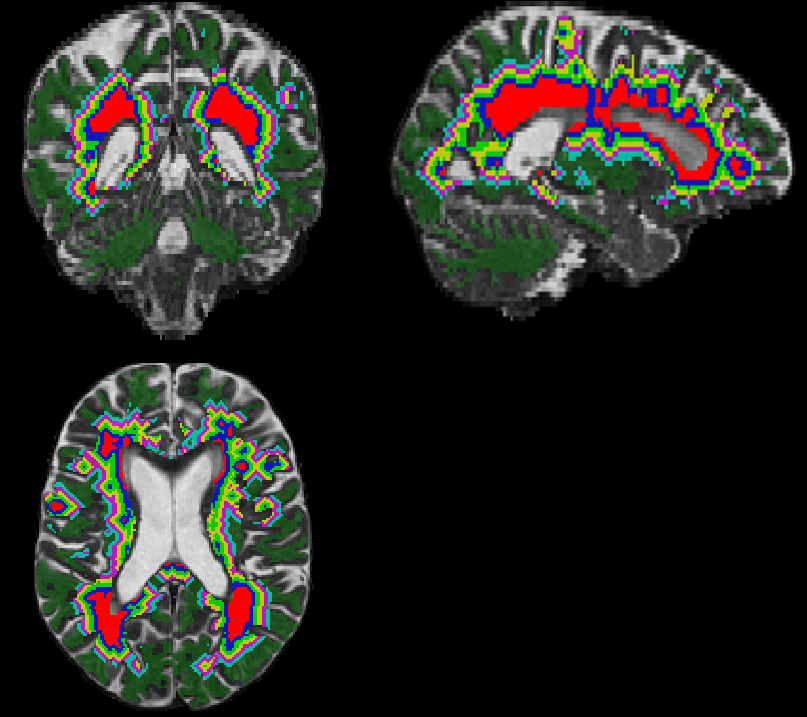

Supplement: Supplementary Material [file mmc1.doc]
